# Multi-Omics Approaches in Immunological Research

**DOI:** 10.3389/fimmu.2021.668045

**Published:** 2021-06-11

**Authors:** Xiaojing Chu, Bowen Zhang, Valerie A. C. M. Koeken, Manoj Kumar Gupta, Yang Li

**Affiliations:** ^1^ Department of Genetics, University of Groningen, University Medical Center Groningen, Groningen, Netherlands; ^2^ Department of Computational Biology for Individualised Medicine, Centre for Individualised Infection Medicine (CiiM), a joint venture between the Hannover Medical School and the Helmholtz Centre for Infection Research, Hannover, Germany; ^3^ TWINCORE, Centre for Experimental and Clinical Infection Research, a joint venture between the Hannover Medical School and the Helmholtz Centre for Infection Research, Hannover, Germany; ^4^ Department of Internal Medicine and Radboud Center for Infectious Diseases, Radboud University Medical Center, Nijmegen, Netherlands

**Keywords:** multi-omics, systems immunology, integrative analysis, immune-related diseases, immune variation

## Abstract

The immune system plays a vital role in health and disease, and is regulated through a complex interactive network of many different immune cells and mediators. To understand the complexity of the immune system, we propose to apply a multi-omics approach in immunological research. This review provides a complete overview of available methodological approaches for the different omics data layers relevant for immunological research, including genetics, epigenetics, transcriptomics, proteomics, metabolomics, and cellomics. Thereafter, we describe the various methods for data analysis as well as how to integrate different layers of omics data. Finally, we discuss the possible applications of multi-omics studies and opportunities they provide for understanding the complex regulatory networks as well as immune variation in various immune-related diseases.

## Introduction

Infections cause millions of deaths each year, and the current COVID-19 pandemic underlines the devastating effects of these communicable diseases. At the same time, the incidence of immune-related diseases such as atherosclerosis (1) and autoimmune diseases such as type 1 diabetes mellitus (2) have been increasing. All these diseases are related to or mediated by the immune system. Thus, the immune system plays a vital role in health and disease, and it is our defense mechanism against harmful substances, infectious diseases and cancer. Within a properly functioning immune system, immune responses should be kept in a certain range, as both hypo-activation and hyper-activation lead to disorders of the immune system. Understanding how the immune system works and what causes the immune system disorders may help us to efficiently fight against immune-related diseases.

However, getting a comprehensive understanding of the immune system is a challenging task. First of all, the immune response is mediated through a complex interactive network of many different immune cells and molecules, such as cytokines, immunoglobulins, and metabolites. At the same time, this network is highly variable depending on the exact threat of the wide variety of pathogens and other substances it’s responding to. To make things even more complex, the immune response to a certain stimulus or infection is highly variable between individuals, leading to population heterogeneity. This heterogeneity is exemplified by differences in severity of patients suffering from the same infectious disease (3), variability in vaccine efficacy (4), and variation in responses to the same medical treatment (5). Many factors contribute to the immune network and the inter-individual variation of immune responses, highlighting both the promise and the challenge of multi-omics studies.

Until now, omics data have been used in many immunological studies to identify the determinants of immune variation and molecular bases of the immune process in different population groups. Properly designed omics studies should make use of appropriate measurements as well as reasonable analytic approaches, which depend on their specific research question. Taking omics studies on COVID-19 as an example, a genome-wide association study revealed eight genetic regions to be associated with critical illness in COVID-19. By integrating both genome and transcriptome data, the authors prioritized one gene, *IFNAR2*, that might play a causal role in COVID-19 (6). Another study, focusing on transcriptome data of immune cells from the lung and blood, identified several pro-inflammatory immune pathways related to the pathogenesis of COVID-19 (7). A proteomics and metabolomics study investigated the changes in COVID-19 patient sera, and identified molecular changes implicating dysregulation in macrophage pathways, complement activation, and platelet degranulation, as well as suppression of metabolic pathways (8). A cellomics and single-cell transcriptome study also revealed dysregulation of the monocyte compartment as well as two neutrophils clusters specific to severe COVID-19 patients (9). Moreover, a study integrating single-cell transcriptome, cellomics, epigenome and proteome comprehensively characterized complex dynamic changes in immune cells. Their results disclose an elevation of IFN-activated megakaryocytes and erythroid cells, hypomethylations around immune signaling genes, and co-expression modules associated with clinical outcome (10). Additionally, a study on fecal fungal microbiota of COVID-19 patients showed enrichment of *Candia albicans* and a highly heterogeneous mycobiome configuration during hospitalization (11). From different angles, these studies make use of omics data to provide insights in the molecular pathology of COVID-19, which can eventually lead to improved therapeutic strategies.

**Figure 1:**
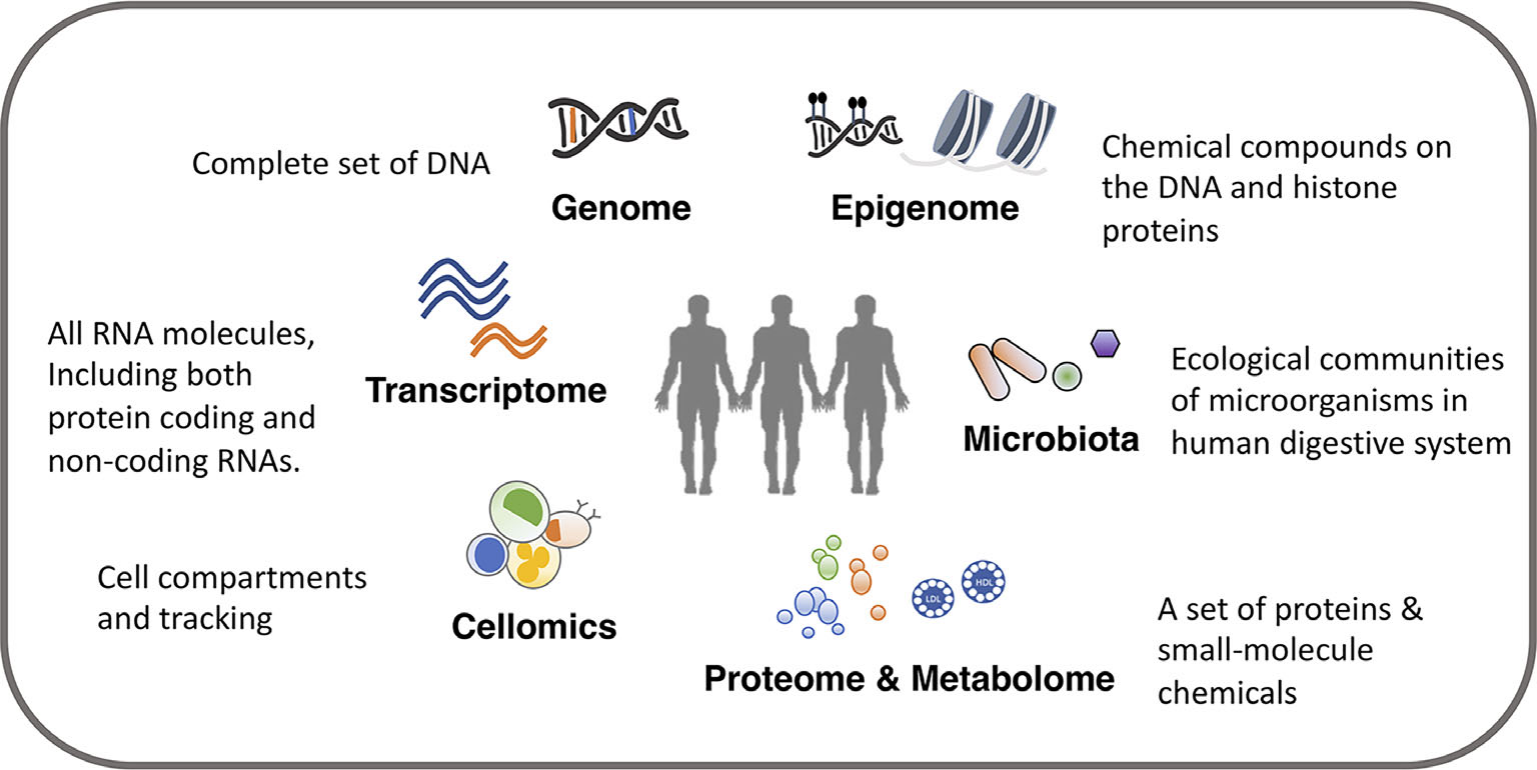
Overview of omics data.

In this review, we present an introduction to multi-omics studies to investigate immune function and variation. The review is split into three parts. In the first section, we describe in brief about the different layers of omics data relevant for immunological research, including genome, epigenome, transcriptome, proteome, metabolome, digestive system microbiota and cellomics (12) [also called cytomics (13)] (**Figure 1**), and the commonly used methodological approaches to measure these different types of omics data. We also discuss important considerations and recommendations for an appropriate study design. In the second section, we discuss how to analyze and integrate multiple omics platforms, including system genetic approaches to identify genetic factors, integration among multiple genetic profiles, as well as the integration and association with other omics data layers. We demonstrate how recent studies applied a multi-omics approach to the immune system researches, and we discuss the interpretation of results from different approaches and their importance in immunological studies. In the third section, we discuss the immunological subjects that need specific attention and may see progress in the next few years. As for detailed information on computational algorithms and models in multi-omics integration (14, 15), imputation on missing omics data (16), and strengths and limitations of system approaches in infectious diseases research (17), we refer readers to other recent reviews.

## Measurements of Omics Data

We can identify potential immunological mediators and study immune phenotypes with a wide range of omics comprising of various molecular and cellular phenotypes including genome, epigenome, transcriptome, proteome, metabolome, digestive system microbiota and cellular phenotypes such as cell composition (**Table 1**). A single omics data layer characterizes a specific biological process from one aspect, for example, transcriptome, but this can only provide insights on genes at a transcriptional level. To achieve a holistic picture of the immune system, a systematic collection of multi-omics data is often required. The tissue (or source) to be measured is another important aspect to be considered. For example, the genome is usually regarded as a stable feature for each individual and collected from an easily accessible tissue, such as blood. Only in some specific contexts, somatic mutations acquired after birth have to be considered and measured in specific tissues (18). However, many other types of omics, such as transcriptome, proteome and metabolome, vary between cell types and tissues. Therefore, it is important to consider the tissue in your experimental design and aim to get as close to the relevant tissue as possible.

**Table 1:** Typical approaches in omics measurements.

	sequencing-based	microarray-based	others
genetics	whole-genome-seq, whole-exome-seq	Illumina OMNI5, Immunochip etc.	–
epigenetics	ATAC-seq, whole-genome bisulfite-seq, RRBS-seq, DNase-seq, FAIRE-seq, ChIP-seq, etc.	MethylationEPIC BeadChip, ChIP-chip, etc.	–
3D chromosome	Hi-C, etc.	–	–
gene expression	RNA-seq, scRNA-seq, SLAM-seq	Affymetrix Genome U133 array, Illumina Whole-Genome Gene Expression BeadChips, etc.	–
protein level	–	–	Immunoassay, MS -based approaches
metabolites	–	–	NMR, MS-based approaches
microbiome	16s rRNA-seq, metagenomics, etc.	–	–
cellomics	single cell sequencing approaches	–	FCM, CyTOF

Given the complexity of the immune system, there is no golden standard for what to collect in multi-omics studies. The necessary data depends on the research question and subjects. Understanding the different layers of omics data is helpful for setting up an appropriate study design. Therefore, in this part, we introduce features and categories of different omics, and describe important considerations when generating these data.

## Genome Variation Measurement

Genotyping detects diversity in the genome. It describes small variations, such as single-nucleotide polymorphisms (SNPs), insertion/deletions (InDels) as well as large-scale mutations such as insertions, deletions and amplifications. Genetic diversity can lead to variation in individual immune function (19).

To date, many techniques can be used for detecting genotypes, including DNA sequencing, DNA microarrays (also known as genotyping chips) and PCR-based methods. These approaches can be categorized based on their measurement scales (high-throughput *vs*. low-throughput methods) or based on whether they include unknown variants (discovery *vs*. screening methods). Classical sequencing-based approaches detect genetic variants in a nearly unbiased manner on the genome (whole-genome sequencing) or within the exome regions (whole-exome sequencing), including known or novel SNPs as well as structural mutation such as short insertions, deletions, and copy number variations.

Considering the cost and effectiveness of genotyping scales and cohort sizes, most of the population-based association studies choose genotyping screening methods, such as DNA microarrays. These methods measure thousands to millions of known SNPs in well-studied organisms, such as humans and mice. The targeted polymorphisms depend on the chip designs. For example, Immunochip contains 196,524 polymorphisms (718 InDels and 195,806 SNPs) on most reported loci involved in autoimmune and inflammatory diseases (20), whereas other custom genotyping chips contain loci designed for specific research areas, such as Metabochip (21) or cardiovascular disease chip (22). The number of variants that can be detected using genotyping chips has increased over the years, but even the high-density 5 million SNPs chip (Illumina OMNI5) covers only a small fraction of the 3.3 billion bases in the human genome.

In order to improve the power in discovering genetic associations on the regions poorly covered by DNA microarrays, genotype imputation approaches are often used to expand the coverage. For example, a commonly used genetic imputation server (https://imputationserver.sph.umich.edu/index.html#)! takes the ~60,000 public available human haplotypes, covering ~40,000,000 SNPs, as a reference to impute millions of missing SNPs based on the measured genotypes and linkage disequilibrium (LD) structures (23).

Before association analysis, genotype data should pass a standard quality control (QC) at both individual level and SNP levels. Individuals with discordant sex information, outlying missing genotype or heterozygosity rate should be excluded (24). Duplicates and relatives could be identified by calculating identity by descent (IBD), and a multi-dimensional scaling plot merging with reference data such as the 1000 Genomes project (25) could help with the identification of individuals of divergent ancestry. SNPs failed in genotyping and/or imputation and SNPs with low frequency and/or that deviate from the Hardy-Weinberg equilibrium are commonly removed before association analysis, especially in array-based studies, because those signals usually relate to bad genotyping quality. However, some SNPs with low frequency may also contribute to rare diseases or phenotypes. With the increase in genotyping quality, more and more recent studies focus on the function of rare alleles (minor allele frequency [MAF] < 0.01) (26–29).

## Epigenome and 3D Chromosome Measurement

Epigenetics describes the study of chromatin traits (either in DNA or histones) that do not involve changes in the nucleotide sequence. Epigenetics measurements are mainly characterized by the changes in histone modification (methylation and acetylation), DNA methylation, chromatin modification, chromatin accessibility, and chromosome structure.

DNA methylation is the process of adding methyl groups to DNA molecules, almost exclusively in CpG dinucleotides with the cytosines on both strands being methylated. This process usually acts in promoter regions to repress gene transcription, and abnormal hypermethylation, which results in transcriptional silencing, is often associated with immune diseases or used as a biomarker (30). Genome-wide techniques, such as whole-genome bisulfite sequencing (WGBS) (31), reduce representation bisulfite sequencing (RRBS-seq) (32) and other non-targeted DNA methylation profiles, provide an opportunity to discover novel biomarkers. Other techniques, such as bisulfite-amplicon sequencing (BSAS) (33) and methylation arrays (34), detect the methylation status of CpG dinucleotides. Similar to genotyping arrays, the targeted regions from methylation arrays are based on the chip design. For the study of the human immune system, some well-established arrays can provide comprehensive coverage. For example, MethylationEPIC BeadChip covers over 850,000 methylation sites, making it ideal for an epigenome association study within big cohorts (35).

As the essential proteins that pack and order the DNA into structural units, histones play a role in gene regulation (36). Histone modification describes the post-translational modifications of histones, including methylation, acetylation and others. Histone methylation often occurs as arginine (R), lysine (K), or histidine (H) residues of histone H3 or H4 being monomethylated (me1), demethylated (me2), or trimethylated (me3). Array-based and sequencing-based approaches, such as ChIP-chip and ChIP-seq (37), are used to identify specific histone modifications that bind to DNA regions or domains.

Chromatin modifications and accessibility is another important aspect of epigenetic changes. One of the most widely-used techniques to capture chromatin accessibility is called Assay for Transposase-Accessible Chromatin using sequencing (ATAC-seq). A standard “bulk” ATAC-seq measurement detects genome-wide open chromatin within a pooled sample or tissue, while in order to capture cellular heterogeneity, single-cell ATAC-seq measures chromatin accessibility in thousands of individual cells, which can generate genome-wide profiles from 10k to 100k cells per experiment (38). Alternative techniques are also used to investigate chromatin phenomena, such as DNase-seq and FAIRE-seq, which measure open chromatin in regulatory regions, MNase, which identifies well-positioned nucleosomes, and ChIP-seq, which is used to detect binding sites of specific transcription factors (39).

Most epigenetic measurements also come with technical errors and biases. Biological replicates and technical replicates can help to characterize variability between samples and sequencing runs. Putting replicates of different conditions in the same batch is also important to avoid batch effects confounding treatment effects. Large projects, such as the Encyclopedia of DNA Elements (ENCODE), have provided standard pipelines for processing many types of epigenetic data, such as ChIP-seq and ATAC-seq. However, this is not applicable in all cases. Applying appropriate QC strategies and software that accounts for bias effects according to the experiment design is essential to obtain robust results. To increase the coverage of epigenetic measurements, several methods, such as ChromImpute (40), Melissa (41), Avocado (42), and SCALE (43), provide imputation approaches for different epigenetic markers. However, the existing imputation approaches have several limitations (16), and are not as widely applied as genotype imputation methods.

3D chromosome structure describes how chromosomes are folded, packaged, and organized into functional compartments, and how different compartments are interconnected. Orthogonal ligation-based approaches include DNA-FISH, which can help with nuclear architecture visualization, and chromosome conformation capture (3C) techniques. One of the 3C techniques, Hi-C, is the most widely used approach to detect interactions between different genome regions (in gigabase-scales) (39, 44). Single-cell adaptation of Hi-C methods are also used to investigate the interactions in individual cells (45).

Ligation-based approaches have the limitation of detecting DNA fragments connected with multiple genomic regions. To overcome this limitation, orthogonal ligation-free methods including genome architecture mapping (GAM) (46), split pool recognition of interactions by tag extension (SPRITE) (47) and chromatin-interaction analysis *via* droplet-based and barcode-linked sequencing (ChIA-Drop) (48) were developed.

## Transcriptome Measurement

The transcriptome comprises all RNA molecules, both coding and non-coding transcripts, in a single or population of cells. Traditional qPCR techniques can only quantify a limited number of genes at the same time. The most commonly used high-throughput techniques are RNA sequencing (RNA-seq) and microarray, and they can detect a large number of genes. Similar to genotyping methods, a sequencing-based approach (RNA-seq) can quantify the entire transcriptome, while microarray-based approaches (e.g., Affymetrix Genome U133 array and Illumina Whole-Genome Gene Expression BeadChips) are designed to target most known genes. In addition, a typical RNA-seq can detect alternative splicing and rare isoforms, which microarray-based techniques cannot.

Certain coverage is required for sequencing data, which depends on the aim of the study. For instance, a bulk RNA-seq study for human differential expression profiling requires 10-25 million reads per sample, while alternative splicing or allele-specific expression analysis need 50-100 million, and identifying novel transcripts requires >100 million reads per sample.

However, a “bulk” like measurement of transcriptome cannot deal with the cell heterogeneity and can be influenced by cell composition changes. Single-cell RNA sequencing (scRNA-seq) was designed to uncover the transcriptome diversity in heterogeneous samples, characterizing the transcriptome in cell resolution. There are several approaches of scRNA-seq, among them are plate-based (Smart-seq2) (49) and droplet-based (10x Genomics) the most commonly used ones. Usually, as few as 10,000 to 50,000 reads per cell are enough to detect cell types, and 500,000 reads can cover most of the genes (50).

In order to increase exonic coverage and accuracy of gene quantification, polyA selection library preparation is commonly applied in scRNA-seq approaches such as 10x scRNA-seq (51). This will, however, miss the important immune repertoire profiling, such as B-cell and T-cell receptors, which is mainly distinguished by their 5’ mRNA sequences. Thus, sequencing facilities, such as 10x genomics, provide full length paired B-cell and T-cell repertoire sequencing, simultaneously, when examining cellular gene expression level. Combined with transcription measurement, this information can improve our understanding of clonal expansion and better characterize immune cell heterogeneity and functions (52).

SLAM-seq detects the newly synthesized RNAs using a metabolic RNA labeling approach. Compared to the other scRNA-seq techniques, this method can track the transcriptome dynamics (53). For example, scSLAM-seq was applied to characterize the onset of infection with lytic cytomegalovirus in single mouse fibroblasts (54).

The transcriptome reflects the dynamic changes in biological processes, which is much more unstable. So, an appropriate sampling strategy on transcriptome data is crucial. In addition to the quality control, normalization is usually performed within a sample and between samples. When considering comparison analysis, it is also necessary to have biological replicates and check for batch effects using clustering-based approaches. There are many computational tools handling batch effects. Of note, integration approaches (55), as included in Seurat (56) and Harmony (57) packages, are commonly used in scRNA-seq analysis which detect the consistent cell type signals from different batches or measurements. However, when the batch difference is confounded with other group information, it will be tough to filter out the batch effects. In addition to batches, it is also important to consider other potential confounders in experiment design. For example, transcriptional differences were observed between males and females in COVID-19 patients (58), thus a gender-balanced design in a case-control study will lead to an unbiased conclusion for COVID-19. Moreover, when considering sampling tissues for immune responses, circulating leukocytes are often measured for systemic inflammatory responses, while inflamed tissues are measured for local inflammatory responses. In order to expand the capacity, deconvolution approaches have been applied to bulk RNA-seq data to characterize cell type compositions (59, 60), while expression recovery methods have been applied to single-cell RNA-seq data to reduce the dropout noise (61, 62). Like imputation approaches in genome and epigenome studies, one should be aware and careful with the potential false signals in these recovery or deconvolution approaches.

## Proteome and Metabolome Measurement

Proteins are the major transcriptional products and functional units in the immune system. Immune molecules like immunoglobulins and cytokines are usually detected and/or quantified by immunoassays such as immunofluorescent staining, enzyme-linked immunosorbent assay (ELISA), enzyme multiplied immunoassay technique (EMIT), or mass spectrometry (MS)-based approaches.

In addition to independent measurement, proteins can be also measured together with RNA transcripts. CITE-seq provides an opportunity of identifying surface proteins along with RNA-seq. This approach is often used for cell labeling in scRNA-seq (63). Cells in different research groups (e.g., under different treatments, from different tissues) could be labeled with different antibodies as hashtags, then sequenced together as one pool. This process has two advantages: decreasing cost and excluding potential batch effects. In addition, as we also know that some immune cell types have specific cell markers, this approach can also be used to identify cell types. For example, the detection of CD3e, CD4 and CD8a proteins on the cell surface could help to distinguish CD4 T cells from CD8 T cells (64). Moreover, there is a new technique called INs-seq, which can measure intracellular protein activity along with scRNA-seq. This new technique shows a large potential of applications in immune-related studies (65).

The study of metabolic processes that regulate immune cell responses, which is referred to as immunometabolism, has become an exciting area in translational research, and is paving the way for novel therapies in immune-related diseases. The intermediate or end products of cellular metabolism are metabolites, which include, but are not limited to, lipids, fatty acids, amino acids, bile acids, and cholesterols. Considering the regulatory effects of metabolites on the immune response (12, 66, 67), the metabolome has become an important subject to study in immunological research.

Approaches to study the metabolome can be classified into targeted and non-targeted techniques. Nuclear magnetic resonance (NMR) spectroscopy is one of the most commonly used techniques, detecting specific nuclei in the target molecule (68). Compared to NMR, mass spectrometry (MS)-based approaches are more high-throughput and quantify metabolites in a non-targeted way, which detect mass-to-charge ratio (69). However, MS-based approaches have a limitation in annotating metabolites, which is the major drawback of this method in contrast to NMR. Metabolites data could be acquired from different sources of samples. Among them, circulating metabolites are the most commonly measured. There are also many studies about fecal and urine metabolites.

Similar to transcriptome analysis, a proper normalization (usually a log transformation) is required in both the proteome and metabolome data process. Secondly, biological replicates and batch effects have to be taken into consideration as well. In addition to linear regression, more advanced computational tools, such as ROIMCR (70), can also be used to reduce the batch effects and to identify metabolites that associate with immune responses. In terms of sampling tissues, in addition to blood cells and inflamed tissues, proteome and metabolome can also be measured in urine, which is thought to be a rich source but underestimated in recent studies (71–73). In addition, fecal metabolites are usually studied together with microbiota, which affects immune homeostasis and susceptibility of the host to immune-mediated diseases. Of note, there is a recent study reporting a reference map for serum metabolites (74), which can serve as a guide to control for irrelevant confounders in serum metabolite studies.

## Digestive System Microbiota Measurement

Microbiota refer to all micro-organisms in a certain environment, for example the human digestive system. It has been reported to vary among individuals, to influence host immune functionality and to be involved in immune-mediated disease pathology (75–77). The commonly used approaches to study microbiota include 16s rRNA sequencing and metagenomics sequencing. After excluding host (human) reads, microbiota reads are aligned to the known microbiome genomes to identify the taxonomies and abundance. While there are also other omics approaches including metatranscriptomics, metaproteomics, and metabolomics, which target transcripts, proteins, or metabolites from microbiota (78).

Of note, studies on human microbiota usually have relatively low concordance compared to other omics data studies. A recent study has reported a number of host variables that could confound human gut microbiota researches. To be exact, body mass index (BMI), sex, age, geographical location, alcohol consumption, bowel movement quality (BMQ), and diet should be balanced in cases and controls when comparing gut microbiota compositions (79). In the context of sample collection, most of the microbiota samples are acquired from the stool, while urine and exhaled gas could be another important resource for microbiome detection (80, 81).

## Cellomics Measurements

Cellomics measurements often reveal the systemic responses at the level of cells and tissues, typically including cell composition, cellular localization and trafficking analyses. Cell composition is measured as cell type abundance or proportion, which is commonly quantified by flow and mass cytometry (82) (FCM and CyTOF) or single-cell sequencing techniques. With the help of cell surface markers or cellular-specific expression markers, both techniques can characterize hundreds of circulating cell subpopulations covering major immune cells involved in innate and adaptive immune responses (i.e., neutrophils, monocytes, lymphocytes, and their subtypes). Additionally, high-content screening (HCS) is commonly used to track cellular changes, including their localization, trafficking and morphologic phenotypes (83, 84).

## Systems Analysis on Omics Data

After data collection and pre-processing with appropriate strategies, the next big challenge lies in linking different omics datasets and clinical phenotypes. For a certain trait or disease, a systems model can be built to specify the role and effect of different data layers. In this model, the qualitative or quantitative characteristics are linked by their relationships, which need to be estimated *via* comparison, association and other systems approaches. These links can simply be a correlation, or a regulatory or causal effect. In this section, we introduce general system approaches among different omics data and provide representative examples of how they can be applied in immunological studies.

## Genome-Wide Association Analysis and Quantitative Trait Locus Mapping

Genome-wide association studies (GWAS) aim to scan the whole genome to find genetic determinants of certain traits. When considering a binary trait (e.g., case-control), we compare allele frequency in two groups of individuals, for example one disease group and one healthy group. A chi-squared test is often applied to test for statistical significance. It is usually considered that there are ~1,000,000 independent loci in the human genome, so a p-value less than the Bonferroni corrected threshold of 0.05/1,000,000 (5 × 10^-8^) is regarded to be genome-wide significant (85).

To date, GWAS have identified ~5000 genetic risk loci of immune-related diseases in ~400 studies (86). Those findings improved our understanding of genetic factors influencing immune-mediated diseases, further pointing to the genetic basis of pathology as well as treatment targets.

Generally, GWAS identify pathogenetic genetic factors contributing to phenotypes (diseases), though those variants will not cause disease directly but affect intermediate molecules. Quantitative trait locus (QTL) analysis is a statistical method to discover the genetic basis of the intermediated phenotypes, such as gene expression (eQTL) (87), splicing (sQTL) (88), metabolites (mQTL) (29), methylation (meQTL) (89, 90), and immune traits (91, 92).

After data normalization, a linear regression between each genetic variant and each quantitative trait is applied. Covariates are crucial aspects of the regression model of QTL analysis. Based on the type of omics, different covariates should be included in the model to correct the detected phenotypes. In general, basic host features such as age and sex are considered, and a population structure has to be additionally taken into account, especially in large cohorts with samples from admixed ancestry (93, 94).

eQTLs are the associations between SNPs and expression of genes, which provide insights of the function of genetic variants. eQTLs can explain 10% - 50% heritability of a phenotype/disease (95), which means that gene expression variation is one of the major consequences of genetic variants. It is very useful for prioritizing pathogenic genes when there is an association between a gene expression and a pathogenic genetic variant. Based on the position, eQTLs are classified into cis-eQTL (eQTL within 1Mb of the gene) and trans-eQTL (eQTL located outside 1Mb of the gene). Among them, trans-eQTLs are more tissue-specific than cis-eQTLs (88). Of note, tissue-specific eQTLs provide a way for prioritizing pathogenic tissues (96).

QTL analysis on epigenome identifies the associations between genetic variants and epigenetic modification. Most genome-wide significant disease-associated loci (~93%) are located in non-coding regions (97), particularly, regulatory elements identified by ENCODE (98) and Roadmap projects (99). These observations highlight the importance of epigenome in the genetic regulation of diseases and immune functionality. Similar to eQTL analysis, this analysis could help us find the potential epigenetic mechanism responsible for the association between genetic variants and immune traits/diseases. For example, a study investigated genetic variants that affect the activity of cis-regulatory domains (aCRD-QTLs) or correlation structure within cis-regulatory domains (sCRD-QTLs) in 317 lymphoblastoid and 78 fibroblast cell lines, and their consequence on gene expression (100). At the same time, genetic variants can also affect methylation (meQTL) by influencing the binding of DNA methyltransferase (DNA MTase). Large meQTL studies in blood samples showed significant enrichment in autoimmune diseases such as ulcerative colitis and Crohn’s disease (101).

pQTL mirrors the associations between genetic variants and protein level. About 40% of cis-protein quantitative trait loci (pQTLs) are also eQTLs, as expected, indicating a sequential genetic regulation between gene expression level and protein levels. By applying pQTL analysis, we could identify the potential mechanism, at the protein expression level, behind the association from genetic variants to immune-related phenotypes. Same as with cis-eQTLs, cis-pQTLs are also located around transcription start sites (TSS). Notably, pQTL showed a significant enrichment on missense, 3UTR and splice region (102). pQTLs could also help with prioritizing causal proteins/genes of immune traits/diseases. For example, a pQTL of serum IL18R1 and IL1RL1 also associates with atopic dermatitis. This association between genetic locus and protein level indicates a possible involvement of IL18R1 and IL1RL1 in atopic dermatitis pathology (102).

Metabolites that mediate the association between genetic variants to immune functionality and immune diseases could be discovered in an mQTL analysis. More than 140 genomic loci are associated with circulating metabolite features explaining a median 6.9% heritability (103). Overlaps between mQTLs and immune traits QTLs suggest the role of metabolic processes in the genetic regulation of immune functionality. For instance, a mQTL study indicates that mQTL loci ARHGEF3 (rs1354034) and LRRC8A (rs13297295) also affect platelet function and neutrophil function, respectively (104).

Immune phenotypes such as circulating immune cell proportion and cytokine production capacity in response to stimulations are crucial parameters when characterizing immune activities. Understanding the genetic determinants of immune phenotypes can provide insights into immune function and immune-mediated diseases. A human functional genomics project has identified >20 genetic factors determining immune cell proportions and cytokine production upon stimulations, which provided a link between genetic control and inter-individual variation (92, 105).

## Integration of Multiple Genetic Association Profiles

In the context of immunological research, multiple diseases, and molecular and cellular phenotypes can be regulated by the same genetic factors, indicating an internal association between them. Integration with multiple genetic profiles can provide insights and build connections between associated phenotypes. Ideally, such genetic profiles can be directly built from GWAS and QTL analysis of different layers from the same individuals. Otherwise, they can be also collected from different population-based cohorts. A number of computational approaches have been developed to discover the link. In particular, approaches like colocalization (106), genetic correlation (107) and Mendelian randomization (MR) (108) take genetic variants as the instrumental variables to infer the association or causality when multiple traits are associated with the same locus.

Colocalization analysis evaluates the association from each of the single locus, and it helps to identify the phenotypes that share the same genetic regulation. Examples of colocalization analysis include a study integrating genetics, epigenetics and transcription to identify colocalization of molecular traits from CD14+ monocytes, CD16+ neutrophils and naïve CD4+ T cells (109). Results from this analysis illustrate molecular mechanism at autoimmune diseases-associated variants, including an alternative splicing signal around SP140 in T cells which might be involved in Crohn’s disease pathology.

Genetic correlation considers the full summary statistics to describe to which extent the genetic background is shared between two phenotypes. An example from a LD regression-based genetic correlation approach showed a shared genetic basis of autoimmune diseases such as Celiac disease and type 1 diabetes (107). This indicates a similar pathological mechanism between these two diseases.

MR is a statistical method working on the step from association to causality. If one trait (exposure) is causal to another trait (outcome), then the genetic factors contributing to the exposure should also contribute to the outcome. This would be reflected in the correlation between effect sizes of the same genetic variant on exposure and outcome. There are many examples of immune-related studies that applied MR, which led to the identification of causal relationships between IL-6 signaling and rheumatoid arthritis (110), IL-18 and inflammatory bowel disease (111) and between eosinophilic indices and asthma (26).

## Comparison and Association of Epigenome and 3D Chromosome Structures

Systems analysis of epigenetic changes can investigate their influence on and changes induced by immune functionality or variation as well as disease susceptibility and development (112, 113). As an example, the impact of cytokines was studied on the epigenome of insulin releasing cells (β cells) from type 1 diabetes pancreases. By measuring ATAC-seq, Chip-seq and RNA-seq, the authors identified proinflammatory cytokines induced neo/primed epigenetic events in human β cells (114). Moreover, in immune systems, the effects of epigenetic changes lead to long-term alterations in the metabolic and transcriptional pathways, and further induce immune memory (115) or immunological diseases (116). Thus, epigenomics is another vital area for better understanding of the personalized immune system.

While genetics is stable, the epigenome is subject to dynamic changes, which can be induced or affected by host and environmental factors, such as smoking, drug usage, diet, aging, inflammation, disease, and exposure to pets. Considering that epigenetic changes affect gene transcription levels, the epigenome is a pivotal part to study when trying to understand immunological networks.

In a case-control study, differential accessible regions (DARs) could be identified in an ATAC-seq data, as well as differential methylation positions/regions (DMP/DMRs) in bisulfite sequencing and methylation array. Instead of comparison analysis, association analysis is applied to continuous phenotypes to get associated regions. Upon the position of acquired regions, we could further map them to the corresponding genes. More specifically, by checking which gene TSS regions are overlapped with the peaks/regions, the peaks/regions could be matched to genes, and then for pathway analysis to get more biological meanings. For example, in a multi-omics study on mixed-phenotype acute leukemia, researchers associated scATAC-seq with transcription responses from scRNA-seq and antibody captured from CITE-seq. Despite widespread epigenetic heterogeneity of chromatin accessibility within patients, they reported common malignant signatures across patients, and thus revealed both distinct and shared molecular mechanisms of mixed-phenotype acute leukemia (117).

Another application of epigenetic analysis is to annotate the function of the identified regions, based on the signals from epigenetic markers. A tool (118) used a multivariate hidden Markov model applied to annotate regulatory elements (e.g., Transcription starting sites, enhancers, promoters) with histone markers (e.g. H3K4me1, H3K4me3, H3K27me3, H3K9me3, H3K36me3) binds to the chromosomes. Applying this method, an example learnt the chromatin states in mice and humans, and reported the up-regulation of immune regulatory regions in Alzheimer’s disease (119).

The analyses on 3D chromosomes are generally similar. In a case-control study with Hi-C data, we could get the compartment switches in a comparative analysis. We could further predict the interactions between those segments (120). Referring public epigenetic databases or genome annotations, we could check the overlap between switched compartments or interactions and known epigenetic markers or elements. Based on this information, we could again associate the changes with other immune profiles or annotate the involved regulatory elements. For example, in a study of lineage commitment of early T cells with Hi-C data, authors found wide compartment re-organizations across chromosomes from a transition between T cell double-negative-2 stage to double-negative-3 stage, and later double-negative-4 stage to double-positive stage. They annotated the changes with domain scores, and more interestingly, they found the changes in the domain scores between the two transitions are positively correlated, which suggests the re-organization at the former transition is actually reinforced at the later transition (121). Another example includes a study on activated T cells, that identified activation-sensitive interactions related to autoimmune diseases captured by Hi-C data (122).

To capture the changes that occur in cellular activation and differentiation, time-series study is another hot topic in associating epigenome and 3D chromosome structures to immune responses. For example, a recent study elucidates the chromosome conformational changes in B lymphocytes as they differentiate and expand from a naive, quiescent state into antibody secreting plasma cells (123). The authors reveal that the changes to 3D chromatin structure occur in two discrete windows, associated with prolonged time in the G1 phase of the cell cycle. Their results also suggest chromosome reconfiguration is linked to a gene expression program that controls the differentiation process required for the generation of immunity.

## Comparison and Association of Transcriptome and Proteome

As the downstream products of genetic and epigenetic regulation, transcriptome and proteome changes directly reflect the influence of genetic and epigenetic variants. Comparison and association studies of transcriptome and proteome have allowed researchers to estimate functional units and validate hypotheses in immune regulation.

As for a case-control study, the first and direct analysis is identifying differentially expressed genes/proteins (DEGs/DEPs), followed by pathway analysis. If the corresponding phenotypes are continuous, then associated genes/proteins will be identified before pathway analysis. Examples include many transcriptome/proteome studies upon the severe infectious disease COVID-19. Transcriptome measurement across samples from healthy, moderate patients and severe patients suggests an overall acute inflammatory response in COVID-19 patients, whereas transcriptional responses of high cytotoxic effector T cells are associated with moderate patients, and deranged interferon responses are associated with severe patients (124). Moreover, a urine proteome study identified 1986 urine proteins showing significant level changes in COVID-19 patients than in healthy controls (125).

Different from bulk RNA-seq, the adding information in scRNA-seq: cell composition, provides more analysis potentials. In a case-control study, in addition to DEGs and enriched pathways identification within each cell cluster/type, cell proportion could be compared between groups while novel cell subpopulation could also be identified in particular cases. For example, a scRNA-seq on two COVID-19 cohorts reported identical dysfunctional neutrophil clusters in severe patients’ blood (9). When considering the TCR/BCR analysis, it would be interesting to explore the clonotype expansion and diversity under different conditions (126, 127), immune development stages (52), or antigen specificity (128). Usually, a clonal expansion means an adaptive immune response targeting certain stimulation, since a certain receptor is the mediator of specific antigen recognition.

Since transcriptome/proteome data is rapidly responding to environmental changes, with the transcriptome/proteome analysis in a time-series study, we could associate the dynamics with infection or stimulation to comprehensively understand the host immune responses. A nice example is demonstrated in a study of influenza vaccination efficiency, where authors measured the hemagglutination-inhibition (HAI) antibody titers and transcriptional responses at baseline and multiple time points post-vaccination. By comparing the profiles between day 28 and day 180, the authors describe individual categories as temporary and persistent responders and illustrate the underneath molecular mechanism (129). Many approaches have been developed for time-series studies, such as regression-based method like maSigPro (130) and a fusion method like O2-PLS (131). Of note, the dynamic study can also be achieved by applying a trajectory analysis such as pseudotime analysis (132, 133) and RNA velocity analysis (134) in scRNA-seq analyses. In a recent study on COVID-19, researchers longitudinally measured samples at several time-point after symptoms, and applied pseudo-time trajectory inference on scRNA-seq data of epithelial cells from the upper respiratory tract. Based on the trajectory, they predicted a new, alternative differentiation pathway that is dependent on the interferon response and marked by interferon-stimulated genes, such as *ISG15*, *IFIT1*, and *CXCL10* (135).

Co-expression analysis among transcriptome or proteome provided information about gene co-regulation and interactions. These co-expression relationships are inferred by different association methods, such as a weighted gene co-expression network analysis (WGCNA) (136) applied on transcriptome to identify consistent expression patterns among genes. The identified associations among gene expression could be applied to predict gene co-regulatory networks, further to prioritize genes involved in the same pathways (137). At protein level, parts of these co-expression relationships could further be explained by protein-protein interactions, which are also collected by several protein-protein interaction databases, including the innateDB (138) who particularly focus on immune interactions. In application, similar to gene co-expression networks, protein-protein interaction relationships could help with functional/pathway enrichment analysis (139).

In the recent single-cell experiments, the co-expression relationships are further applied to predict the cell-cell interactions. By detecting the correlation between known ligand and receptor genes among different cell sub clusters, we could infer the potential communications between cell populations (140). This analysis fits well with immune network analysis. For example, by detecting ligand and receptor genes signals, a recent study identified cross-talks between CD8+ T cells and epithelial cells altered in the colon of ulcerative colitis patients compared to healthy controls (141). Additional methods, such as NicheNet (142), also take knowledge of gene regulatory networks or protein-protein interaction networks from public databases and literatures, then build a model to further predict the activated targets of the cell-cell interactions by correlating the ligands expression level with its potential downstream gene or protein level interactions. In an example study of cell-cell interaction underlying the tissue-specific imprinting of macrophages, the authors deciphered the interaction signals driving monocyte recruitment, engraftment, and acquisition of the Kupffer cells associated transcription factors, and they identified the contributions of different cells to Kupffer cell niche (143).

## Comparison and Association on Metabolome/Microbiome

Metabolome or microbiome are additional factors that reflect, or affect, a person’s state of health (144, 145). Similar to transcriptome or proteome, comparison and association analysis could be applied on metabolome and microbiome data. However, metabolome can be hardly linked to genes, which leads to different strategies of interpretation. Taking KEGG (146) and HMDB (147) as references, an online tool MetaboAnalyst performed metabolic pathway enrichment and network analysis on the identified metabolites (148). An example serum study on COVID-19 detected accumulation of 11 steroid hormones and suppression of amino acid metabolism in patients (8)

As for the gut microbiome, a diversity analysis could be applied to taxonomy data. There are different strategies available for functional profiling on the gut microbiome data. For example, HUMAnN takes metagenomic or metatranscriptomic sequencing data as input to identify gene families and abundances (149). Gene families could be further matched to broader functional categories, such as MetaCyc metabolic pathways and GO categories for functional interpretation. For example, a study associated gut microbiome features to cytokine production capacity, and found microbial metabolic pathways: palmitoleic acid metabolism and tryptophan degradation to tryptophol showed associations with TNFα and IFNγ production (150).

As in transcriptome and proteome analyses, time-series studies could provide valuable information in metabolome and microbiome data. For example, in a study of metabolic functions of gut microbes from patients with Inflammatory Bowel Diseases, fecal samples were collected at baseline and 2, 6, and 14/30 weeks after induction of therapy to collect metabolic and microbiota profiles. The observed association in dynamics of metabolites and diversity shifts of microbiota reveals the heterogeneity of the disease, and helps the authors to build a silico model that might be used to identify patients likely to achieve clinical remission from the therapy (151).

## Integration of Epigenome, Transcriptome, Proteome, Metabolome, Microbiota and Cellomics

Besides associations between omics data and genetics, a simple association analysis between two different non-genetic omics data could be applied to the data measured in the same cohort with a large sample size to find the co-regulations behind (**Table 2**). For instance, eQTMs (associations between methylation and gene expression) provide a resource to integrate methylation and gene expression. Highly methylation can block the binding of transcription factors on promoters and enhancers. In line with expectation, most eQTMs showed negative correlations between methylation and gene expression, and negatively correlated eQTMs are enriched in active TSS regions (152). For another example, a study carefully characterizes the changes in the gut microbiota of patients suffering inflammatory bowel diseases and the interplay between microbiome composition and gut metabolites (153).

**Table 2:** System analysis between omics.

	binary traits	epigenetics	gene expresion	protein level	metabolites	microbiome	cellomics
genetics	GWAS	meQTL, CRDQTLs	eQTL, sQTL	pQTL	mQTL	mbQTL	cell proportion QTLs
epigenetics	DMRs/DARs/Compartment Switches/Gained or lost Interactions	position-based overlap	gene-based overlap/association	gene-based overlap/association	association	association	association
gene expresion	DEGs	–	co-expression	gene-based overlap/association	association	association	association
protein level	DEPs	–	–	coexpression/interaction	–	association	association
metabolites	different abundance	–	–	–	association	association	association
microbiome	different composition	–	–	–	–	association	–
cellomics	Different cell composition, etc.	–	–	–	–	–	association

In the situation of a more complex multi-omics integration, more advanced technique like building multivariable regression model could take features from different omics to evaluate the accumulative effects/prediction power on a certain phenotype. An example study integrates genomic, metagenomic, metabolomic, immune cell composition, hormone levels and platelet activation profiles with cytokine response profiles in a population-based cohort. Results from multivariable linear regression and machine learning approaches such as elastic net show the accumulative contribution and predict power of genetic and non-genetic factors on cytokine response (154).

On the other hand, if the sample size is not allowed for association analysis, it might be applicable to check the intersections between the findings from different omics. For example, we could easily compare the regions identified in ATAC-seq, methylation array and Hi-C data. In addition, by matching a DAR to genes, and intersecting with DEGs, we could further check whether an epigenetic change has the potential in regulating gene expression.

## Discussion and Perspectives

**Figure 2:**
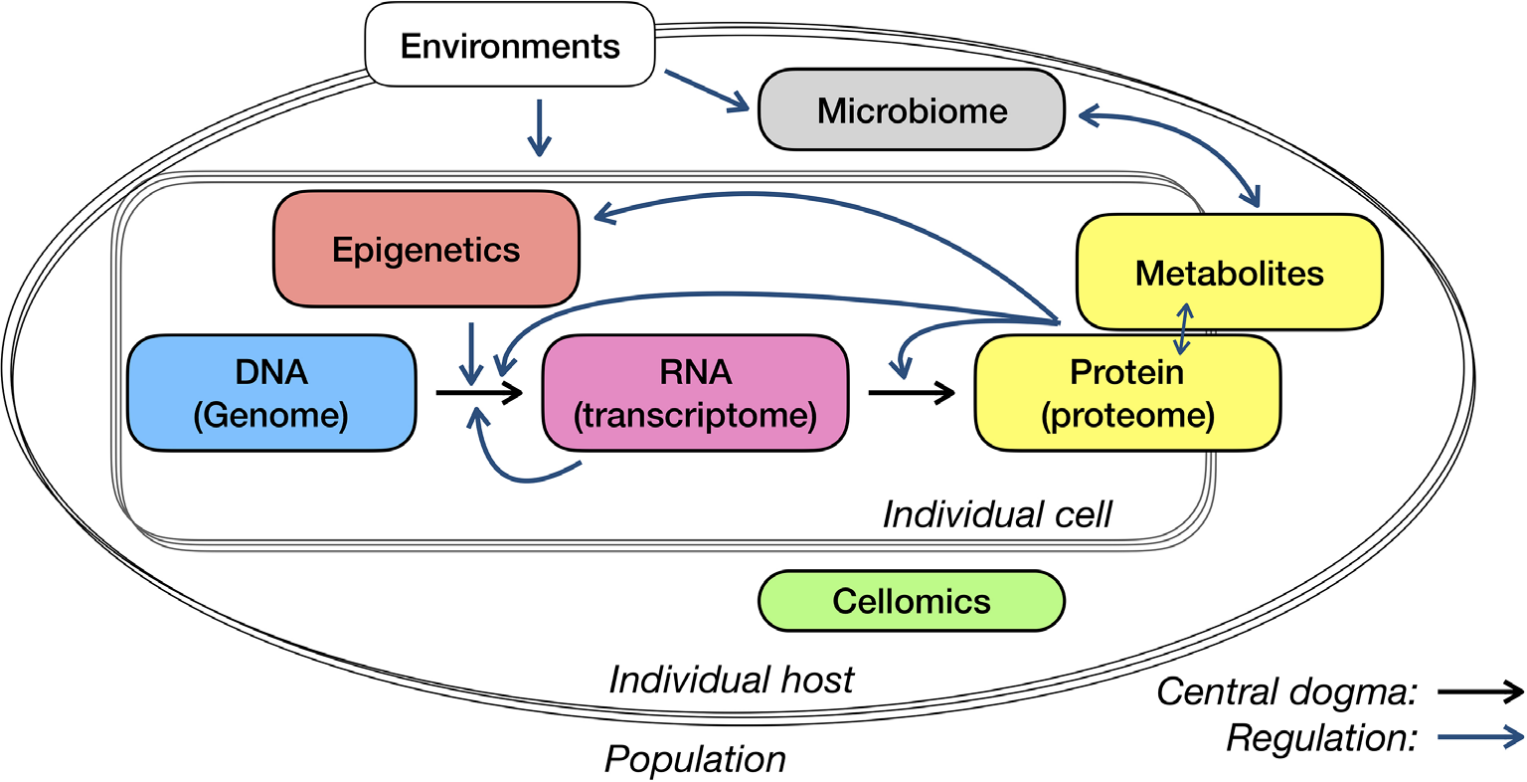
Central dogma and regulations of different omics layers.

In this review, we have discussed the multi-omics application for immunological studies, from measurements and analysis to comparison or association of several typical layers (**Figure 2**). For system studies – in particular newly discovered infectious diseases or rare diseases with fewer prior knowledge – the choice of data layers to collect and the selection of measuring approaches, target or non-target technique, bulk or single-cell level, can be as important as the analysis models and algorithms. Here, we discuss a few points that need specific attention in study design and interpretation, and subjects may see progress in the next few years.

There are some commonly used strategies of interpreting genetic associations. As the starting point of the central dogma of molecular biology (**Figure 2**), genetics has so far received a lot of attention and was associated with many types of data or phenotypes. In the interpretation of genome-wide associated loci, genes around them have also been regarded as the necessary and most essential compartments. The strategy to properly link loci with affected genes so far has been addressed on the position and associations between gene expression and genetic variants (i.e., eQTLs). In addition, functional annotation on identified loci, such as whether the variants are located on the regulatory elements or affected protein structure, may provide additional clues for loci interpretation in particular cases. Nevertheless, there are existing debates upon several aspects, for example, whether the host genome could influence the gut microbiome. It will never be nitpicking to be very careful with interpreting your microbiome QTLs.

Epigenetic could be used as a window to study environmental influence. In contrast to genetics, epigenetics often links the external factors to immune phenotypes. This is particularly true when considering the external effects as a risk to immune diseases, for example, smoking to asthma, because epigenetic modifications, such as methylation, are usually related to environmental exposures. Considering the various kinds of epigenetic changes, multiple types of epigenetic data are commonly used in one study and they often validate and complement each other. For example, an active TSS region could be identified by low methylation as well as high DNA accessibility (155), and the enhancer involved in a neo chromatin interaction identified in Hi-C data could be characterized as a neo opening region in ATAC data (156). Considering the functional relationships, epigenetic data is commonly integrated with gene expression measurements. As the direct consequence of epigenetic modification, alteration in corresponding gene expression could be the best validation of the importance of your epigenetic studies.

scRNA-seq is usually applied together with Cellomics measurements. A cell composition discovered in scRNA-seq data could be validated with FCM-based approaches. FACS is also commonly used as a pre-filtering step to help with concentrating target cell types for scRNA-seq analysis. Especially, for the rare cell types (e.g., T regs in PBMCs), a pre-sorting process is necessary for concentrating on cells of interest.

Proteome, metabolome showing downstream immune functions require more attentions. As the downstream products of gene expression, protein or metabolites level measurements are not as popular as transcriptome measurements in current studies. This might because gene expression analysis takes advantage of the efficiency of next-generation sequencing and well-established microarray chips. Thus, there appears to be much room for further studies on proteome and metabolome in immune studies.

Proper measurement techniques and sampling tissues are crucial in an omics study. When considering the purpose of measurements, it is often appropriate to apply high-throughput and/or non-target approaches at the discovery stage, while single and/or target approaches are more commonly used for validation. Besides, except genome, all the other omics have tissue specificity. Data from the same tissue are more commonly associated. For example, associations between omics from blood samples could be easily interpreted, but it would be tricky and needs more biological basis to associate blood features with gut features.

A straight-forward joint visualization of multi-omics data is another challenge to better present and understand the interconnections across molecular layers as well as to fully utilizing the increasingly available omics data. Integrated tools or platforms that combined a comprehensive analysis workflow and interactive visualizations were often more preferable to researchers. Some examples are: PaintOmics3 (157) and Metascape (158), which provide powerful online frameworks for the multi-omics pathway analysis and visualization; Seurat (56), which focuses on analysis and visualization of single-cell omics data and supports easy connections to other popular analysis tools; and Omics Playground (159), who provides a user-friendly and interactive self-service bioinformatics platform for analysis, visualization and interpretation of transcriptomics and proteomics data. Moreover, trials of combing data sharing and interactive visualization along with research publication have also been made to improve the data dissemination. For example, by accessing to Immgen (160), FastGenomics (161) or DeCovid (58), researchers can explore and visualize their interested immune signatures on the COVID-19 datasets, which significantly increases impact of the studies.

To fully elucidate the biological processes involved in the immune system, several aspects remain unknown in omics studies. Firstly, due to sample accessibility, fewer studies have been performed on tissues other than blood. Taking meQTLs as an example, several big studies have been carried out blood samples (101, 162, 163). However, there are very limited sample size and/or studies about meQTLs in other tissues (164). Secondly, considering the high dynamics, rapid response and spatial specificity of the immune system, temporal and spatial studies can provide more insights into the dynamic process and spatial heterogeneity in immune activities and/or immune-related diseases etiology. For example, the process that immune cells are activated by interacting physically and chemically with synapses is highly dynamic and depends on the spatial position of immune cells, neurons and glial cells. Despite its importance in immune functionality and immune-mediated diseases, our current knowledge is not sufficiently advanced, which calls for more comprehensive studies (165–167). Thirdly, as for population-based studies, there are much more of them in healthy individuals of European ancestry, while the studies in under-represented populations as well as in patients appeal for greater attention.

Considering the complexity of our immune system and patient heterogeneity, in terms of severity or treatment responses, for many immune-related diseases, the generation of personalized medicine is one of the most significant goals we can achieve through multi-omics studies (168). Personalized medicine stratifies a heterogeneous group of patients based on certain characteristics and provides treatment based on this stratification. In the case of infectious diseases, one of the personalized medicine trials is now being conducted for the treatment of sepsis using immunomodulatory interventions after stratification based on biomarkers identifying immunosuppression or hyper inflammation (169). In the field of tuberculosis, advances are being made too, as a clinical trial is now ongoing where tuberculous meningitis patients are being stratified based on genotype prior to treatment (170).

In conclusion, we systematically review measurements and analyses can be applied in immunological studies, which provide insights for personalized medicine. Through the development of high throughput techniques, e.g. single-cell RNA sequencing and mass cytometry, we now possess the tools to unravel the many complexities of the immune system in health and immune-related diseases, including infectious diseases, allergies and auto-immune diseases. With unbiased measurements and effective integration, multi-omics studies can help us understand the immune system and could lead to the development of personalized medicine.

## Author Contributions

XC made the conception and design of the review. XC and BZ drafted the manuscripts, supervised by YL. All authors contributed to the article and approved the submitted version.

## Funding

XC was supported by the China Scholarship Council (201706040081). YL was supported by an ERC Starting Grant (948207) and the Radboud University Medical Centre Hypatia Grant (2018) for Scientific Research.

## Conflict of Interest

The authors declare that the research was conducted in the absence of any commercial or financial relationships that could be construed as a potential conflict of interest.
